# Azastilbene Analogs as Tyrosinase Inhibitors: New Molecules with Depigmenting Potential

**DOI:** 10.1155/2013/274643

**Published:** 2013-02-12

**Authors:** Larissa Lavorato Lima, Rebeca Mól Lima, Annelisa Farah da Silva, Antônio Márcio Resende do Carmo, Adilson David da Silva, Nádia Rezende Barbosa Raposo

**Affiliations:** ^1^Departamento de Química, Instituto de Ciências Exatas, Universidade Federal de Juiz de Fora, Campus Universitário, 36036-900 Juiz de Fora, MG, Brazil; ^2^Núcleo de Pesquisa e Inovação em Ciências da Saúde (NUPICS), Universidade Federal de Juiz de Fora, Campus Universitário, 36036-900 Juiz de Fora, MG, Brazil; ^3^Faculdade de Odontologia, Universidade Federal de Juiz de Fora, Campus Universitário, 36036-900 Juiz de Fora, MG, Brazil

## Abstract

This research has been an effort to develop synthetic resveratrol analogs in order to improve the depigmenting potential of natural resveratrol. Six resveratrol analogs were synthesized and tested for tyrosinase inhibitory activity *in vitro*, by qualitative and quantitative steps. The results showed the analog **C** as being the most powerful tyrosinase inhibitor (IA_50_ = 65.67 ± 0.60 **μ**g/mL), followed by the analogs **B**, **E**, **F**, **A**, and **D**, respectively. The analog **C** presented a tyrosinase inhibition potential better than natural resveratrol (*P* < 0.001). The best depigmenting activity was provided by the presence of hydroxyl in the orthoposition on the second phenolic ring.

## 1. Introduction

Animal and human skin color is mainly determined by the content of melanin pigment. Although melanin has a mainly photoprotective function in human skin, the accumulation of an abnormal amount of melanin in different parts of the skin resulting in more pigmented patches might become an aesthetic problem [1]. Such conditions may appear due to numerous factors, including sun exposure [2], genetic factors [3], pregnancy [4], diseases [5], use of certain medicines, and others [6].

**Figure 1:**
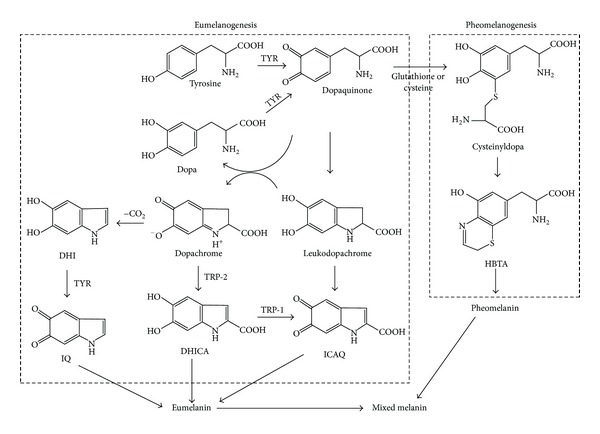
Melanin biosynthetic pathway [27]. TYR: tyrosinase; TRP: tyrosinase related protein; DOPA: 3,4-dihydroxyphenylalanine; DHICA: 5,6-dihydroxyindole-2-carboxylic acid; DHI: 5,6-dihydroxyindole; ICAQ: indole-2-carboxylic acid-5,6-quinone; IQ: indole-5,6-quinone; HBTA: 5-hydroxy-1,4-benzothiazinylalanine.

Tyrosinase (phenol oxidase) is known to be a key enzyme implicated in the anabolism of melanin biosynthesis in melanocytes [7, 8]. This enzyme catalyzes two different reactions: the hydroxylation of monophenolic compounds to *o*-diphenols and the oxidation of the *o*-diphenols to *o*-quinones. The enzyme converts tyrosine to 3,4-dihydroxyphenylalanine (L-DOPA) and oxidizes L-DOPA to form dopaquinone, which plays a prominent part in melanin biosynthesis [6, 7] (Figure 1). The inhibition of the enzyme tyrosinase has been the subject of many studies [6–10]. 

The compounds utilized in the treatment of hyperpigmentation usually act as either competitive or noncompetitive inhibitors of tyrosinase, thereby blocking reaction steps of the pathway shown above, and consequently, blocking melanin synthesis [6, 7, 11]. Among the skin-lightening and depigmenting agents, magnesium-L-ascorbyl-2-phosphate, hydroxyanisole, *N*-acetyl-4-S-cysteaminylphenol, arbutin (hydroquinone-beta-D-glucopyranoside), salicylhydroxamic acid, dioic acid, kojic acid, and hydroquinone are the most widely used in the cosmetic industry, being prescribed worldwide [8, 12–14]. However, there are reports on potential mutagenicity and epidemics of ochronosis, as well as on adverse reactions that may aggravate the appearance of the spots and damage the health of patients who make use of such agents [6, 12].

The use of hydroquinone, for instance, has been associated with a number of adverse effects, including skin irritation, contact dermatitis, and exogenous ochronosis in dark-skinned people [15, 16]. Other commonly available topical agents, such as corticosteroids, are either less effective or more likely to cause local or systemic side effects after long-term use [17]. Moreover, the use of cosmetics containing hydroquinone is prohibited in the European Union and is strictly controlled in the United States by the Food and Drug Administration (FDA) [18]. Instead, dioic acid has been used to treat hyperpigmentation, with good efficacy, but with similar side effects as hydroquinone [19]. For that reason, there has been an increasing impetus to find alternative herbal and synthetic pharmaceutical depigmenting agents [6, 12, 17].

Resveratrol (3,5,4′-trihydroxystilbene), a natural molecule found in many plants, including mulberries, peanuts, and principally grapes [7], has been considered in previous studies due to its pharmacological properties such as antioxidant, anti-inflammatory, antiaging, cardioprotective, neuroprotective, and anticancer effects [20–23]. Resveratrol and its hydroxyl derivatives have also been presented as inhibitors of mushroom tyrosinase [6, 7, 24]. However, some studies have demonstrated that resveratrol does not inhibit the synthesis of melanin to such a degree that enables it to be utilized alone as skin whitening agent in pharmaceutical formulations, and so its use as an adjuvant in hyperpigmentation treatments is suggested [6, 7, 25].

**Figure 2:**
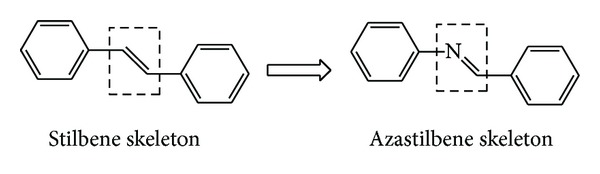
Comparison of the basic structures of stilbene and azastilbene skeletons.

In a recent effort, our research group has been developing the synthesis and biological evaluation of synthetic resveratrol analogs, particularly the azastilbenes and bioisosteres of natural stilbenes (Figure 2), seeking to improve the potential of the resveratrol analogues. Such compounds have shown good antioxidant activity [23, 26] which encouraged us to test the potential of this class of compounds in other areas, such as depigmenting action. Thus, this paper presents a study of the *in vitro* inhibitory activity of six other resveratrol analogs in tyrosinase activity.

## 2. Materials and Methods

### 2.1. Samples

**Scheme 1:**
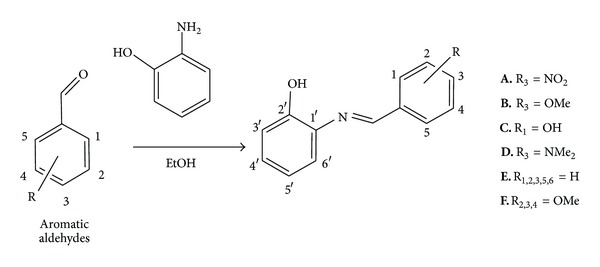
Synthetic pathway for resveratrol analogs.

Azastilbene analogs (**A**–**F**) were synthesized by means of condensation between 2-hydroxyaniline with a variety of aromatic aldehydes in ethanol (Scheme 1). All compounds were characterized by ^1^H and ^13^C nuclear magnetic resonance (NMR), infrared (I.R.), and melting point (M.P.) (Table 1) and were in accordance with data in the literature [28–32].

### 2.2. Preparation of Samples

The six azastilbene analogs were dissolved in 25% dimethyl sulfoxide (DMSO) aqueous solution to obtain solutions with concentrations between 35–350 mg·mL^−1^, used in the tyrosinase inhibition activity assay. The resveratrol (RV03.081F6, Attivos Magisttrais, São Paulo, Brazil) was tested in parallel with the test substances for comparison purposes.

### 2.3. Test for Tyrosinase Inhibitory Activity

The ability to inhibit the activity of the tyrosinase enzyme was evaluated using the enzymatic method described by Macrini et al. [10] with modifications. This method relies on the inhibition of tyrosinase in the presence of its substrate tyrosine, interrupting the melanin synthesis.

#### 2.3.1. Tyrosinase Inhibition Qualitative Enzymatic Reaction Screening

Aliquots of 10 *μ*L of a solution composed of 125 U·mL^−1^ of mushroom tyrosinase (Sigma-Aldrich, USA) were added to 96-well microplates, and then 70 *μ*L of pH 6.8 phosphate buffer solution and 60 *μ*L of the substances (350 *μ*g·mL^−1^, in 2.5% DMSO) were also added. For positive control, 60 *μ*L of kojic acid (17.5 *μ*g·mL^−1^ in 2.5% DMSO) was used instead of the test substances, and for negative control, 60 *μ*L of 2.5% DMSO were added. To the resultant mixture, 70 *μ*L of L-tyrosine (Sigma-Aldrich, USA) at a concentration of 0.3 mg mL^−1^ in distilled water were added (final volume in the wells = 210 *μ*L). 

The absorbances of the microplate wells were read in a microplate spectrophotometer reader (SpectraCount, Packard, USA) at 510 nm (*T*_0_). Then, the microplates were incubated at 30 ± 1°C for 60 minutes and the absorbances were registered again (*T*_1_). An additional incubation for 60 minutes at 30 ± 1°C was done and after this period a new spectrophotometric reading was conducted (*T*_2_).

The inhibitory percentage at the two times (*T*_1_ and *T*_2_) was obtained according to the following formula for inhibitory activity percentage:

(1) $$\begin{array}{c} \text{I}{\text{A}}_{\%}=\frac{C-S}{C}\times 100, \end{array}$$

where IA_%_ = inhibitory activity; *C* = negative control absorbance; and *S* = sample or positive control absorbance (absorbance at time *T*_1_ or *T*_2_ minus the absorbance at time *T*_0_).

#### 2.3.2. Tyrosinase Inhibition Quantitative Enzymatic Reaction Assay

For samples which reached an IA_%_ greater than 50%, a quantitative assay was conducted. With this purpose, the above experimental protocol was followed, with modifications (a 500 U·mL^−1^ tyrosinase solution was used instead of the 125 U·mL^−1^ and the absorbances were measured every 10 minutes, for 1 hour).

The quantitative determination was obtained through an analytical curve and its respective linear equation. For that, the analogs were diluted in microplate wells to 5 concentrations between 35 and 350 *μ*g·mL^−1^ with 25% DMSO, and the kojic acid was diluted to concentrations of 10, 5, 2.5, 1.25, and 0.625 *μ*g·mL^−1^. Samples were assayed intriplicate. The analytical curve was plotted between the tyrosinase inhibition activity percentage at each time and the concentrations of the analogs/positive control. The inhibitory activity at 50% (IA_50_, in *μ*g·mL^−1^) was calculated by using linear equation.

### 2.4. Statistical Analysis

A descriptive statistical analysis and ANOVA followed by the Tukey *post hoc* test were performed, with the Statistical Package for Social Sciences (SPSS) v.14.0 for Windows software, to compare the average values obtained between the resveratrol analogs and the resveratrol analogs versus positive control (kojic acid) standard. The level of significance was *P* < 0.001.

## 3. Results and Discussion

The results obtained by the tyrosinase inhibitory ability assay demonstrated that all the analogs presented IA_%_ greater than 50% in screening (qualitative assay). In the first hour of the assay, the analogs **A**, **B**, **C**, **E**, and **F** presented the same capacity to inhibit tyrosinase compared with kojic acid (*P* = 0.116, *P* = 0.895, *P* = 0.002, *P* = 0.045, and *P* = 0.936, resp.) while in the second hour of the assay all the analogs analyzed and the resveratrol showed better capacity to inhibit tyrosinase than kojic acid (*P* < 0.001). 

Regarding the quantitative assay, all the analogs and the resveratrol showed 4 tyrosinase inhibitory capacity statistically lower than the kojic acid standard (*P* < 0.001). In comparison with resveratrol, the analog **C** was the one which presented the best AI_50_, while analog **D** was the one which presented the worst AI_50_ (*P* < 0.001 for both). The analogs **A**, **B**, **E**, and **F** presented AI_50_ statistically similar to resveratrol (*P* = 0.177, *P* = 0.001, *P* = 0.217, and *P* = 0.999, resp.). All results are shown in Table 2.

All the analogs showed a more stable IA_%_ (capacity to keep their depigmenting ability in the first hour until the second hour) in qualitative assay compared to kojic acid. Phenolic compounds, such as the analogs tested, form relatively stable intermediates because of the resonance of the aromatic ring present in their structure [33]. Similar results were found by Franco et al. [6] who studied the depigmenting activity of phenolic compounds (azastilbene analogs); three analogs (*n* = 6) showed inhibitory ability statistically equal to kojic acid in the second hour of the qualitative assay (*P* > 0.05). However, the compounds studied by Franco et al. [6] have a structural difference when compared with those evaluated in this study due to the existence of an additional hydroxyl group at 2′-position of **A** ring, which characterizes the originality of our work. 

The best tyrosinase inhibition potency was found in analog **C** (IA_50_ = 65.67 ± 0.60 *μ*g/mL) which has an insertion of a hydroxyl in ortho position, C6′, on the second phenolic ring. It presented depigmenting ability better than resveratrol (*P* < 0.001). However, it was statistically different from kojic acid (*P* < 0.001). The presence of a hydroxyl group in such position of the aromatic ring thus appears to be critical for good tyrosinase inhibitor activity. This is an expected result since resveratrol has hydroxyl groups in its structure which are directly linked to its antioxidant activity, as described in the literature [23, 34]. 

In sequence, the analogs **A** (IA_50_ = 95.57 ± 0.74 *μ*g/mL), **B** (IA_50_ = 70.60 ± 1.22 *μ*g/mL), **E** (IA_50_ = 79.03 ± 1.78 *μ*g/mL), and **F** (IA_50_ = 88.80 ± 0.44 *μ*g/mL) presented AI_50_ statistically similar to resveratrol (*P* = 0.177, *P* = 0.001, *P* = 0.217, and *P* = 0.999, resp.). All of them were statistically different from kojic acid (*P* < 0.001).

Analog **B** has a methoxy in paraposition (C4′) while analog **F** has three methoxy groups in *meta-* (C3′ and C5′) and para*-* (C4′) positions, which demonstrates that the inclusion of methoxy groups was not interesting to improve tyrosinase inhibition potency, as was also noted by Franco et al. [6]. The analog **E**, without substituent in the aromatic ring, continued presenting depigmenting potential statistically similar to analogs **B** and **F** (*P* = 0.180 and *P* = 0.085, resp.) and to resveratrol (*P* = 0.217). Finally, analog **A** presented an insertion of a nitrogroup in paraposition, C4′, which may be able to chelate metals and block the action of tyrosinase due to unshared pair of electrons in their molecular structure that is able to complex with copper [6, 35]. This happens because tyrosinase is a copper-protein enzymatic complex that requires copper ions to promote the redox reactions, essential in production of melanin [35, 36]. However, it was not enough to improve the tyrosinase inhibition potency of said analog.

The analog **D** showed worse AI_50_ than resveratrol and kojic acid (*P* < 0.001 for both). 

It demonstrated that the insertion of an amine in paraposition, C4′, was the least interesting modification made in this study for depigmenting action [33]. Franco et al. [6] observed that the analog with insertion of disubstituted amine on the base molecule was not an interesting modification for the quantitative tyrosinase inhibitory activity assay.

Among the six compounds evaluated in this work, three of them (**A**, **B**, and **D**)—which do not owe hydroxyl group at the ortho-position of the ring and present more lipophilic groups—have been previously synthesized [6] and exhibited similar depigmenting activity. However, the compounds **C**, **E**, and **F** present a significantly different structure and, during the assay, a good tyrosinase inhibition potency was observed, being that **C** and **E** were even better than resveratrol (*P* < 0.001 for both).

Although only six analogs have been analyzed in this study, it is possible to observe that molecules with polar groups, such as hydroxyl, confer a higher tyrosinase inhibitory activity than the analogs with fewer polar groups such as methoxyl, nitro, and amine, highlighting the importance of a polar substituent to the molecule.

The above results showed that skin depigmenting and lightening agents must continue to be the subjects of extensive research due to their easy availability and vast clinical results. Different types of compounds from both natural and synthetic sources with depigmenting activity deserve further investigation. Moreover, such agents for skin whitening have a great potential in the cosmetics industry, as they are considered to be safe and largely free from adverse side effects. However, more concrete studies with a human clinical point of view are required [12].

## 4. Conclusions 

The number and position of substituents seem to play an important role in the inhibitory effects of azastilbene compounds on tyrosinase activity. According to the data, the presence of hydroxyl in the ortho position was the molecular modification which gave the best evaluated tyrosinase inhibitory activity. 

Despite the fact that the tyrosinase inhibitory activity was lower than that of the reference standard tested (kojic acid), the azastilbene compound (analog **C**) with orthohydroxylation presented better depigmenting activity (lower IC_50_) as well as when compared with the natural compound (resveratrol), which indicates that said molecule may have pharmacological utility in a near future. Studies on *in vivo* efficacy and tissue biocompatibility of such analogs must be performed to make them feasible for therapeutic use in skin whitening treatments.

## Figures and Tables

**Table 1:** Spectral data of resveratrol analogs.

Compounds	*δ*CH=N	*δ* C=N	$\overset{-}{\nu }$C=N	Melting point (°C)	Yield (%)
**A**	8.90	157.18	1625.8	160.0–161.0	78.0
**B**	8.62	158.46	1622.0	90.1–92.0	66.0
**C**	8.96	160.77	1631.6	149.0–150.3	90.0
**D**	8.50	158.84	1614.3	118.0–119.0	50.0
**E**	8.69	159.73	1625.8	90.8–91.5	83.0
**F **	8.63	158.87	1623.9	118.7–119.6	72.0

*The NMR experiments were performed at 300 MHz for ^1^H and 75 MHz for ^13^C in dimethyl sulfoxide (DMSO-*d*_6_) (ppm) and I.R. experiments were performed at KBr support (cm^−1^).

**Table 2:** Tyrosinase inhibitory activity of new molecules and kojic acid.

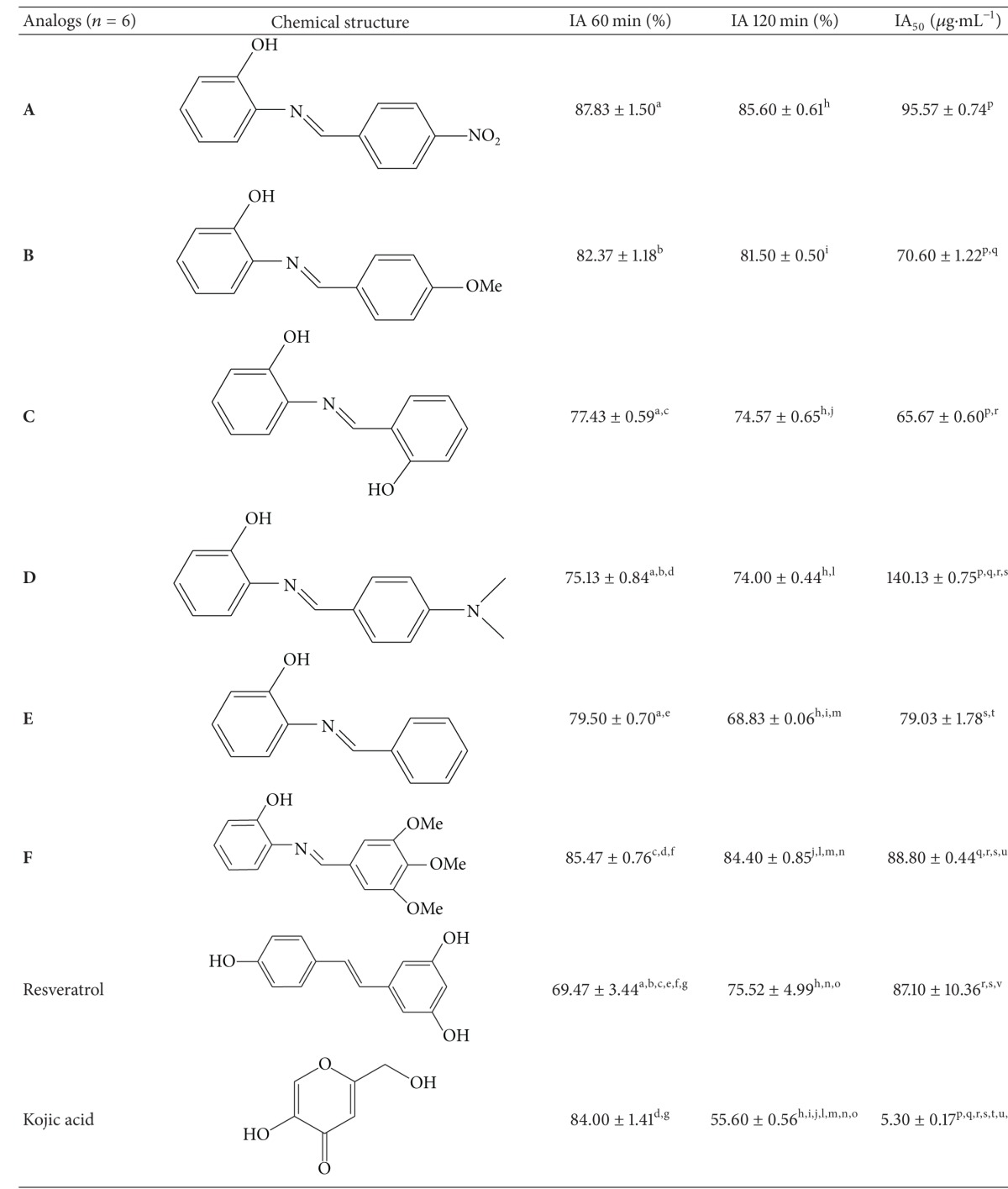

*Means followed by the same letters differ by ANOVA followed by Tukey* post hoc* test (^a–v^ *P* < 0.001).
